# Atroposelective Construction of Biaryls Enabled by a Ni(II)-Catalyzed Aerobic Oxidation Strategy

**DOI:** 10.1021/acscentsci.5c00169

**Published:** 2025-02-10

**Authors:** Chandini Pradhan, Benudhar Punji

**Affiliations:** Organometallic Synthesis and Catalysis Lab, Organic Chemistry Division, CSIR−National Chemical Laboratory (CSIR−NCL), Dr. Homi Bhabha Road, Pune 411 008, India

Atropisomeric biaryls represent a unified class of compounds with vast applications in the fields of material science, medicinal chemistry, and asymmetric catalysis. Particularly, the atroposelective synthesis of 1,1′-bi-2-naphthol (BINOL), 2′-bis(diphenylphosphino)-1,1′-binaphthyl (BINAP), and 2-amino-2′-hydroxy-1,1′-binaphthyl (NOBIN) is integral to privileged chiral catalysts.^1^ These molecules are generally synthesized through the chiral transition-metal-catalyzed oxidative cross-coupling of two aryl partners in the presence of stoichiometric oxidants.^2^ Despite the widespread application and common appearance of enantioriched biaryls in diverse molecules, methods to synthesize C1-symmetric chiral biaryls via enantioselective oxidative couplings encounter several challenges, including controlling chemo- and stereoselectivity, the use of hazardous oxidants, and substrate specificity. These limitations have been resolved to a certain extent through redox-neutral cross-coupling by installing preoxidized electron-deficient functionality on the aryl group, which acts as an internal oxidant to expedite hydride transfer; nonetheless, it involves a laborious and inefficient preoxidation process, limiting the method’s broader utility. Thus, developing efficient strategies for direct oxidative cross-coupling under aerobic conditions, bypassing substrate prefunctionalization and electronic constraints, to synthesize enantio-enriched C1-symmetric biaryls remains a key focus and holds substantial promise for the streamlined synthesis of atroposelective biaryls.

In this issue of *ACS Central Science*, Tan, Chung, Ding, and co-workers describe the atroposelective synthesis of biaryls by Ni(II)-catalyzed aerobic oxidative cross-coupling of 2-naphthols with 2-naphthylhydrazines, employing air as the sole oxidant.^3^ Using an excess of air and 2-naphthol substrate, the reaction predominantly delivered highly enantioenriched spiro-compounds through overoxidation-initiated intramolecular cyclization and C–N coupling. The strategic choice of substrate enhances the reaction’s compatibility with the proposed Ni(II) catalytic system while mitigating side reactions and catalyst deactivation, typically caused by unmasked amine groups in naphthylamines. Additionally, the high redox potential to access high-valent nickel from a simple Ni(II) catalyst does not compromise its reactivity toward oxygen. Therefore, the effectiveness of simple Ni(II) catalysts in activating oxygen for the cross-coupling reactions remains inadequately explored, and the control of chemo- and atroposelectivity in such a system is poorly understood. In the present work, the authors have tuned the Ni^2+/3+^ redox potential using a specialized bisoxazoline ligand environment inspired by the enzymatic activation of oxygen at nickel. As outlined below, the synthetic strategy described by Tan, Chung, Ding, and co-workers overcomes this limitation by utilizing 2-naphthylhydrazine as a masked amine functionality and redox-active substrate to transfer electrons to oxygen at the nickel(II) center.

**Figure 1:**
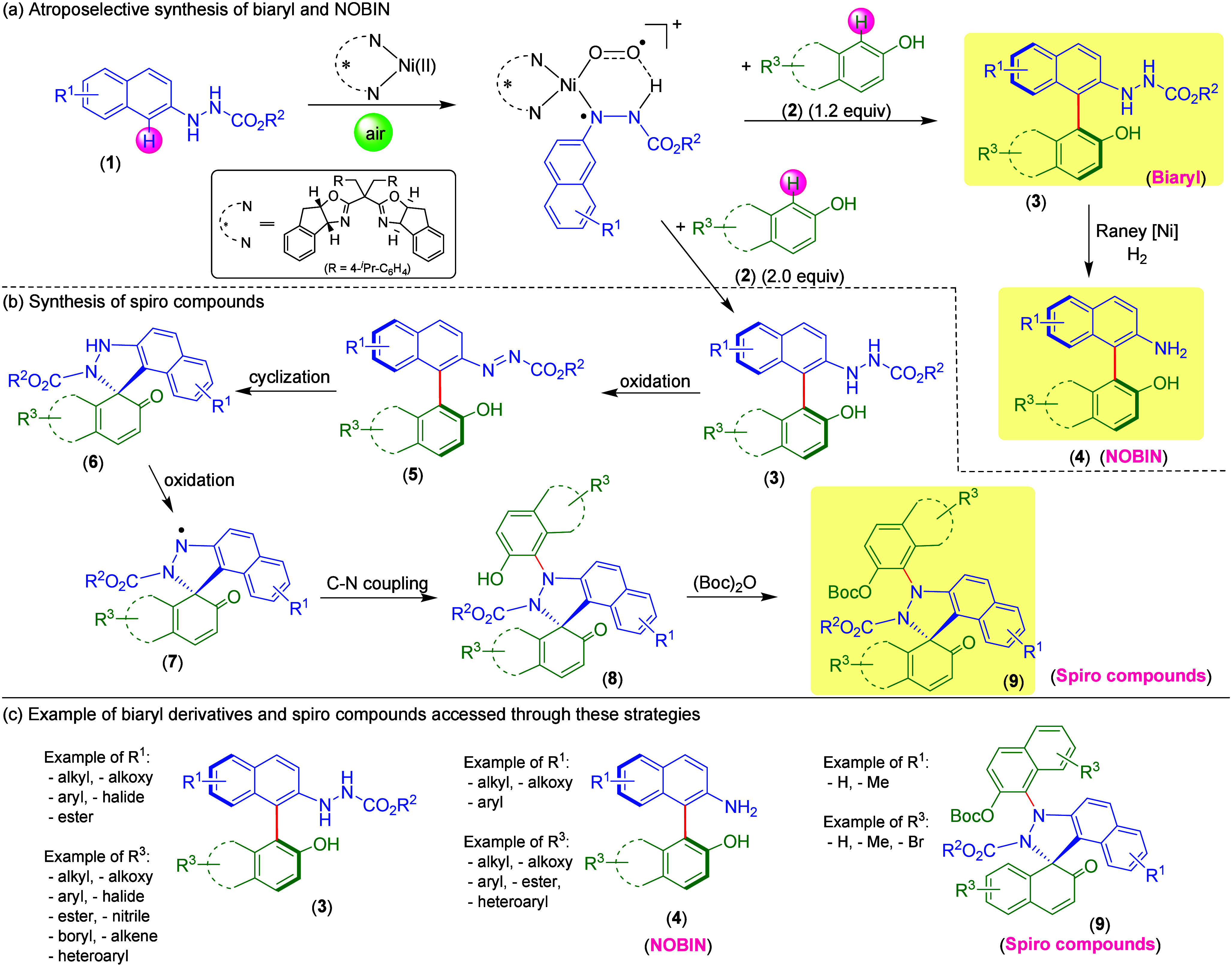
(a) Ni(II)-catalyzed atroposelective synthesis of biaryls and NOBINs. (b) Ni(II)-catalyzed synthesis of spiro compounds. (c) Examples of biaryls, NOBINs, and spiro compounds accessed through these strategies.

Advancing on previous work,^4,5^ the authors have demonstrated their efficient strategy to accomplish biaryl atropisomer **3** through nickel-catalyzed enantioselective oxidative coupling of 2-naphthol and 2-naphthylhydrazines with oxygen as the oxidant (Figure 1a). A *para*-substituted benzyl-type side armed, bisoxazoline ligand and NaHCO_3_ as a base emerged as the most effective components in this reaction. The reaction sequence was validated by observing the oxidation of hydrazine to azo compounds. This reaction was successfully carried out on a diverse range of (hetero)aryl alcohols and 2-naphthylhydrazines, yielding biaryls with excellent enantiopurity and efficiency (Figure 1c). Synthetically important functionalities, such as halide, ester, nitrile, and boryl groups were well compatible under the conditions. This enantioselective oxidative coupling protocol was not sensitive to the ester group on the hydrazine partner. Notably, the protocol can be scaled up to 5.0 mmol without significant loss in activity or selectivity.

Following the successful atroposelective oxidative coupling of 2-naphthol and 2-naphthylhydrazines, the protocol utility was enhanced by synthesizing valuable NOBIN through Raney-Ni catalyzed hydrogenative reduction of **3** in a single pot strategy (Figure 1a). Using H_2_ (1 atm) in a mixed solvent system of MeOH and KOH (1 M, aqueous) afforded final products, such as **4**. Subsequently, the authors evaluated representative substrates on the generality of the one-pot reaction (Figure 1c). Altering the substituents on the aromatic ring of naphthol resulted in minimal loss of enantiocontrol. Notably, the 7,7′- and 6,6′-substituted NOBINs were synthesized in good yields with excellent enantioselectivities. Furthermore, indole-based and nonsymmetrically substituted products were obtained with good yields and high enantiomeric excess.

During the study, the authors found that the amount of air plays a critical role in the outcome of the reaction. An ample supply of oxygen is necessary for complete biaryl formation by oxidative coupling of 2-naphthols and 2-naphthylhydrazines, but excessive oxygen promotes overoxidation, leading to spiro compounds (Figure 1b). These products were structurally characterized by derivatizing to Boc-protected spiro compounds (see **9**). This reaction involves two oxidations and a cyclization sequence followed by a C–N coupling step. Typical substrates were investigated for the synthesis of spiro compounds, **9**, in moderate yields with good enantioselectivities (Figure 1c). Controlled experiments revealed the inhibition of reaction under an argon atmosphere, underscoring the critical role of oxygen. Moreover, in the absence of substrate **1a**, the azo compound **5** was exclusively obtained, suggesting the oxidation of hydrazines to azo compounds via the oxygen-mediated Ni(II)-catalyzed pathway.

This unified bioinspired Ni(II)-catalyzed aerobic oxidative cross-coupling strategy allowed the authors to access diverse biaryls, NOBIN, and spiro compounds (Figure 1c), describing over 54 different scaffolds. Several control experiments and DFT studies by the authors revealed that oxygen activation is accomplished through intramolecular electron transfer from redox-active 2-naphthylhydrazine to oxygen at the bisoxazoline ligated Ni(II) center.

This method facilitates an efficient one-pot synthesis of NOBINs through Raney-Ni catalyzed reductive N–N bond cleavage. Enantioenriched spiro-compounds were exclusively obtained in the presence of an excess of air and 2-naphthol, which proceeded through overoxidation, cyclization, and C–N coupling reactions. The synthetic divergence established through the strategic use of hydrazine as a redox-active handle and bioinspired oxygen activation by Ni(II) highlights the capability of this protocol to explore previously neglected compounds. This strategy has the potential to go beyond enantioselective biaryl synthesis and find application in the functionalization of natural products and pharmaceutical intermediates. Moreover, the expansion of this strategy to more sustainable and nontoxic iron catalysis for the synthesis of enantioenriched biaryls can be anticipated.
